# Ecological niche differentiation in *Chiroxiphia* and *Antilophia* manakins (Aves: Pipridae)

**DOI:** 10.1371/journal.pone.0243760

**Published:** 2021-01-13

**Authors:** Mariana Villegas, Bette A. Loiselle, Rebecca T. Kimball, John G. Blake

**Affiliations:** 1 Department of Wildlife Ecology and Conservation, University of Florida, Gainesville, Florida, United States of America; 2 Center for Latin American Studies, University of Florida, Gainesville, Florida, United States of America; 3 Department of Biology, University of Florida, Gainesville, Florida, United States of America; Federal University of Mato Grosso do Sul, BRAZIL

## Abstract

Species distribution models are useful for identifying the ecological characteristics that may limit a species’ geographic range and for inferring patterns of speciation. Here, we test a hypothesis of niche conservatism across evolutionary time in a group of manakins (Aves: Pipridae), with a focus on *Chiroxiphia boliviana*, and examine the degree of ecological differentiation with other *Chiroxiphia* and *Antilophia* manakins. We tested whether allopatric sister species were more or less similar in environmental space than expected given their phylogenetic distances, which would suggest, respectively, ecological niche conservatism over time or ecologically mediated selection (i.e. niche divergence). We modeled the distribution of nine manakin taxa (*C*. *boliviana*, *C*. *caudata*, *C*. *lanceolata*, *C*. *linearis*, *C*. *p*. *pareola*, *C*. *p*. *regina*, *C*. *p*. *napensis*, *Antilophia galeata* and *A*. *bokermanni*) using Maxent. We first performed models for each taxon and compared them. To test our hypothesis we followed three approaches: (1) we tested whether *C*. *boliviana* could predict the distribution of the other manakin taxa and vice versa; (2) we compared the ecological niches by using metrics of niche overlap, niche equivalency and niche similarity; and (3) lastly, we tested whether niche differentiation corresponded to phylogenetic distances calculated from two recent phylogenies. All models had high training and test AUC values. Mean AUC ratios were high (>0.8) for most taxa, indicating performance better than random. Results suggested niche conservatism, and high niche overlap and equivalency between *C*. *boliviana* and *C*. *caudata*, but we found very low values between *C*. *boliviana* and the rest of the taxa. We found a negative, but not significant, relationship between niche overlap and phylogenetic distance, suggesting an increase in ecological differentiation and niche divergence over evolutionary time. Overall, we give some insights into the evolution of *C*. *boliviana*, proposing that ecological selection may have influenced its speciation.

## Introduction

The distributional area of a species is an expression of its evolutionary history and its ecology [1,2]. Therefore, predictive models of species’ geographic distributions are not only useful for identifying ecological characteristics that may limit a species’ range [3] but are also useful for setting the stage to infer patterns of speciation [4,5]. Species distribution models can be combined with phylogenies to study ecological divergence and evolution of niches, and therefore allow for inferring how were the processes responsible for the formation of new species (e.g., [6–9]). Speciation history should leave a detectable signature in present-day phylogenetic patterns and also in current species geographic distributions [4,10].

The niche is often discussed as either a fundamental or a realized niche. A fundamental niche is defined by the set of abiotic conditions where a species potentially is able to persist, whereas the realized niche describes the conditions in which a species actually persists given the presence of competitors or predators [11,12]. Species may retain aspects of their fundamental niche over long periods of time, a process often called niche conservatism [12]. We can use the present-day ecological niche of a species in a comparative way to help understand the evolutionary history of a species and, potentially, modes of speciation. In general, it has been hypothesized that if ranges of sister taxa do not overlap, the mode of speciation is allopatric; whereas if sister taxa co-occur, sympatric speciation is inferred [5]. In allopatric speciation, new lineages arise after geographic separation of ancestral species into isolated sets of populations [13]. Especially for recently diverged species, if speciation is allopatric, sister species will display little or no overlap in geographic range [4]. Further, we might expect species that differentiated via allopatric modes to retain aspects of their fundamental niche (niche conservatism) [12]. In contrast, species that have differentiated in sympatry may be expected to have diverged in their ecological requirements, and such ecological differences may have driven speciation. Although such species may still share aspects of an ancestral climate niche, they might be kept apart by selection against hybridization, or they might have evolved different climate niches [14]. In summary, speciation is a process in which species’ ranges may expand or contract in response to several factors (e.g., climate, degree of specialization, dispersal capabilities, etc.) [15].

Here, we use species distribution models and niche comparisons to test the hypothesis that ecological niches are conserved across evolutionary time. Our goal was to understand the coarse-scale ecological and geographic properties of species’ distributions [16,17] and, as a consequence, we most closely follow the Grinnellian niche concept (which focuses on the set of coarse environmental conditions for a population to persist, [18]). To test this hypothesis of niche conservatism, we used manakins (Aves, Pipridae) as a model clade. Manakins are an ideal model because they are remarkably diverse, they are broadly distributed across different habitats in the Neotropics, they are well represented in museum collections [19], and are relatively well known with respect to biogeography and speciation [20,21].

**Fig 1:**
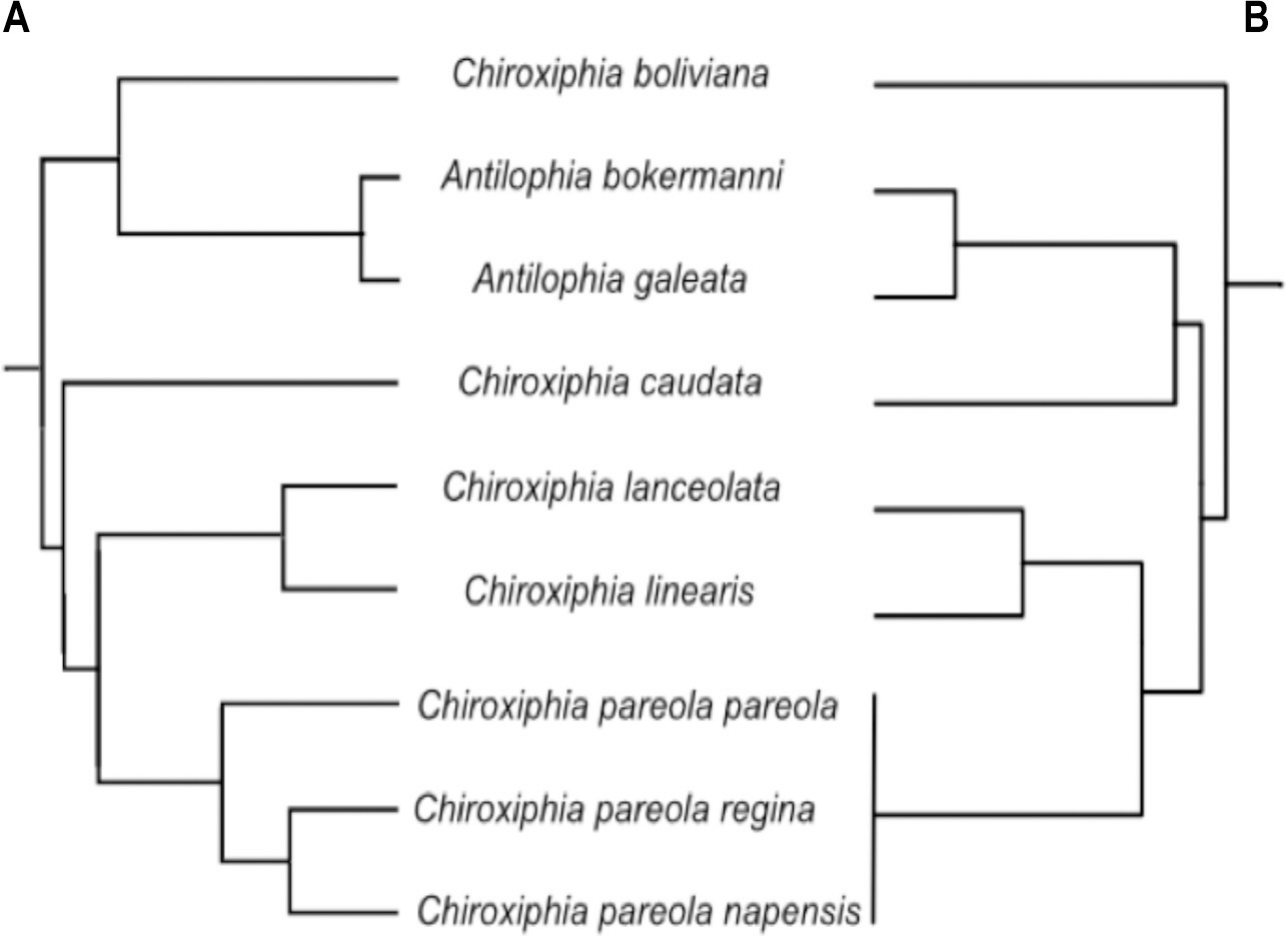
Topologies from molecular phylogenies of *Chiroxiphia* and *Antilophia*. (A) Phylogeny by Silva et al. [25], and (B) Phylogeny by Leite et al. in revision (the molecular phylogenies were kindly shared by the authors).

We focus on the sister genera *Chiroxiphia* and *Antilophia* [22] which form a distinct clade apart from most other genera of manakins [23–25]. The genus *Chiroxiphia* comprises five species: *C*. *linearis*, *C*. *lanceolata*, *C*. *pareola*, *C*. *caudata* and *C*. *boliviana*; *Antilophia* comprises two species: *A*. *galeata* and *A*. *bokermanni* (endemic to a tiny area in the Brazilian northeast) [24]. *Chiroxiphia* has been regarded as a superspecies, with multiple closely related taxa separated geographically [24]. All these characteristics make *Chiroxiphia* and *Antilophia* ideal models to test hypotheses of ecological niche differentiation and speciation. There are two recent studies (Fig 1) that performed molecular phylogenies considering all species in these genera and the results of both suggest that *Chiroxiphia* is paraphyletic ([25]; Leite et al. in revision); while the first one indicates that *C*. *boliviana* is the sister taxa to *Antilophia*, the latter suggests its sister taxa might instead be *C*. *caudata*.

A study on ecological niches of the whole family Pipridae [19] reported niche conservatism between sister species. They did, however, recognize some exceptions, including *Chiroxiphia boliviana* and *Chiroxiphia pareola*; they showed that most manakins have a lowland distribution and suggested that *C*. *boliviana* might have invaded higher elevations and cooler climates from humid lowlands, the latter of which is a more characteristic habitat of the genus. Given that *C*. *boliviana* reaches the highest elevations in the family, here we focus on this species. The present study extends the approaches followed by Anciães and Peterson [19] in four main ways: we focus on *C*. *boliviana* in comparison with other *Chiroxiphia* and *Antilophia* manakins, making it a more detailed approach; we significantly increase the sample sizes of all taxa; we add robust methods to describe and compare the ecological niches in environmental space; and finally, we perform a correlation between niche and phylogenetic distance.

Specifically, the overall goals of this study were to test hypotheses of niche conservatism and speciation in *C*. *boliviana*, with other *Chiroxiphia* and *Antilophia* manakins. We first use species distribution models to identify the environmental variables that best describe *C*. *boliviana*’s ecological niche and compare its niche with those of other *Chiroxiphia* and *Antilophia* manakins; we then test the hypothesis that ecological niches in these genera are conserved across evolutionary time. We followed the framework developed by Graham et al. [5], which examines geographic ranges of species and their environmental envelopes in a phylogenetic context as a way to explore factors that may have influenced speciation. According to this framework, if allopatric sister species segregate in environmental space more than expected given phylogenetic distance, it suggests their niches are not conserved over time, and that ecologically mediated selection may have had a role in speciation. Alternatively, if allopatric sister species are very similar in environmental space, their ecological niches may be more conserved over time than expected, suggesting that ecological divergence (in relation to the parameters examined) has not been a major factor in speciation [5]. To test the hypothesis raised above, we followed three approaches: 1) we examined whether the other closely related manakin taxa could predict the distribution model developed for *C*. *boliviana*; and as well, whether *C*. *boliviana* could predict the distribution models developed for other closely related manakin taxa; 2) we compared the ecological niches of all manakin taxa considered using the following niche metrics proposed by Broennimann et al. [26]: overlap, equivalency and similarity; and finally 3) we assessed whether the ecological niches changed more or less than expected based on phylogenetic distances.

## Materials and methods

We include a checklist (S1 Table) to describe the details for the species distribution models we conducted; we followed guidelines by Feng et al. [27].

### Species occurrence data

We obtained occurrence data for the following manakin taxa with Central and South American distributions: *Chiroxiphia boliviana*, *C*. *pareola* (three of its subspecies were treated separately: *C*. *p*. *pareola*, *C*. *p*. *regina* and *C*. *p*. *napensis*), *C*. *caudata*, *C*. *lanceolata*, *C*. *linearis*, *Antilophia galeata* and *A*. *bokermanni*. Occurrence data included personal sources (personal observations for *C*. *boliviana* and *C*. *pareola regina*), records kindly provided by M. Anciães (for *A*. *galeata*, *C*. *caudata*, *C*. *lanceolata* and *C*. *linearis*), I. Areta (several records for *C*. *boliviana* in southern Bolivia and northern Argentina), J. P. Gomez (several records for *C*. *lanceolata* in Colombia), some records from specimens deposited at the Colección Boliviana de Fauna (CBF) not reported in other museums, and records from citizen science and natural history museums available on the internet through e-bird, GBIF (Global Biodiversity Information Facility, http://www.gbif.org) and ORNIS (provider institutions included: Kansas University Natural History Museum, Macaulay Library, Yale University Peabody Museum, American Museum of Natural History, Smithsonian Institution, Louisiana State University Museum of Natural Science and Cornell Lab of Ornithology; accession dates: December 2015 and January 2016; see S1 Table for more details). Location data were first mapped on ArcGIS 10.4 to inspect for georeferencing errors and to avoid duplication; we also discarded obvious misplaced localities [28]. In general, we tried to use only records that were at least 1 km apart, to reduce sampling bias; however, in few cases we used record locations that were closer because we wanted to have a complete representation of each species’ range. In total, we used 542 records (temporal range of records: 1871–2016): 66 records for *C*. *boliviana*, 16 for *C*. *pareola napensis*, 17 for *C*. *pareola regina*, 74 for *C*. *pareola pareola*, 146 for *C*. *caudata*, 93 for *C*. *lanceolata*, 81 for *C*. *linearis*, 40 for *Antilophia galeata*, and 9 records for *A*. *bokermanni*. This study is the most exhaustive conducted so far, in terms of geographic representation for these genera. Anciães and Peterson [19,29] used less than 10 occurrence locations to model the distribution of *C*. *boliviana*, for example. They also considered all subspecies of *C*. *pareola* together; however, these subspecies are different in body size and to a lesser extent in male coloration and they have relatively well-defined geographic distributions, typically with separation by rivers [24]. In the recent study conducted by Silva et al. [25], they used multilocus DNA sequences from all species and subspecies of *Chiroxiphia* and *Antilophia* to infer phylogenetic relationships, and they found two divergent clades within one of the subspecies (i.e., *C*. *p*. *pareola*) on the northern and southern sides of the Amazon river. Given the substantial differences among subspecies, they could be actual separate species (i.e., especially *C*. *p regina* and *C*. *p*. *napensis*; [25]), hence our decision to treat them as separate units for analyses.

### Environmental data

Initially, we considered 23 environmental variables to define ecologically suitable locations for our study species. These variables included: 16 bioclimatic variables that described annual and seasonal temperature and rainfall trends (WorldClim version 1.4, [30]), three that described topography (slope, eastness and northness obtained from DIVA-GIS; [31]) and four that described vegetation (derived from NDVI–[Normalized Difference Vegetation Index] taken as a measure of the reflectance of Earth’s surface vegetation and representative of leaf area index; [32]). Environmental conditions, especially temperature and rainfall, are major determinants of species distributions at macroscales, and remotely-sensed indices such as NDVI, can complement and improve niche models [33]. Eastness and northness were obtained after transforming aspect; these variables go from a scale of 1 (east and northward, respectively) to -1 (west and southward, respectively). We used NDVI data from 9 years, January 2005 to December 2013, downloaded from the Copernicus Global Land Service program (available at http://land.copernicus.vgt.vito.be/PDF/portal/Application.html#Home). These NDVI measurements were derived from satellite-borne remote sensors (Top of Canopy SPOT/VEGETATION and PROBA-V data). We used the maximum and minimum monthly values out of the 9 years to calculate the following vegetation layers: overall maximum NDVI, overall minimum NDVI, mean annual NDVI and coefficient of variation NDVI. Accession and download from these sources were done in January 2014. All the environmental variables were in raster format and were prepared in ArcGIS 10.3 to align in geographic space using a WGS84 datum system, and to match in spatial extent and cell size (~1 km^2^ cell size, or 0.00833 decimal degrees); following preparation, environmental layers were converted to ASCII raster format for later spatial analyses.

To reduce the number of environmental variables, we followed methods by Parra et al. [32] and plotted 1,000 random points within the geographic study area (from southern Mexico to northern Argentina), extracted the associated environmental variables and with these values performed a correlation matrix with these values (S2 Table). To reduce multi-collinearity, we removed variables that had a coefficient of correlation > 0.8 with other environmental variables (S2 Table). Thus, we used 13 environmental layers to construct the species distribution models (SDMs): annualpp (annual precipitation), maxtwarmmo (maximum temperature of warmest month), meantdryqua (mean temperature of driest quarter), ppcoldqua (precipitation of coldest quarter), ppdryqua (precipitation of driest quarter), ppwarmqua (precipitation of warmest quarter), ppseason (precipitation seasonality: standard deviation *100), tseasoncv (temperature seasonality), overall maximum NDVI (maxndvi), coefficient of variation NDVI (cvndvi), eastness, northness and slope. We selected these variables because they have been shown to be important for the ecology of bird populations. Precipitation variables (i.e., the amount and timing of rainfall) significantly affect the demography, survival and abundance not only of Neotropical birds [34] but also of tropical rainforest Australian birds [35]. Similarly, seasonality in both temperature and precipitation were fundamental in determining the phylogenetic composition of hummingbird communities in Ecuador [36]. Further, climate variables together with vegetation productivity variables such as NDVI, have proved to be important determinants of seasonal niches of long-distance migratory birds [37].

### Species Distribution Models (SDMs)

All SDMs were performed in Maxent 3.3.3 [38] (version 2013) which uses the principle of maximum-entropy (finds the distribution that is closest to uniform) to estimate a set of functions that relate environmental variables and habitat suitability to estimate a species’ potential distribution [39]. Maxent is designed to work well with presence-only data, has a high performance with small datasets [38,40] and has been tested extensively and proven to be a robust machine-learning technique [41,42, S1 Table].

Models were run using the default regularization values (i.e. regularization penalizes the use of too many model parameters; it forces Maxent to focus on the most important features by avoiding overfitting; [38,41]). We also chose the logistic model output. The logistic model output is a transformation of the relative occurrence rate, which describes the relative probability of presence [43]; it is a continuous surface of values ranging from 0 to 1 (i.e., high values indicate a high probability of occurrence; [44]). Given that specificity (i.e., proportion of cells correctly predicted as absence cells in relation to all absence cells) cannot be calculated with presence-only data, a threshold of predicted probability was selected: the resulting models were converted to presence-absence using a 10th-percentile training presence threshold (this identifies the top 90% of training samples; [45]). With the resulting rasters, we used ArcGIS 10.7 to make maps of the discrete and continuous relative suitability ranges of each species. For these maps, we also used two layers: a global country boundaries layer [31] and a Digital Elevation Model (DEM) raster [46].

To develop models for each species and subspecies, we first randomly partitioned each species’ data (occurrence locations) into two data sets: 75% used as training data (i.e., to formulate the model parameters) and 25% as test data (i.e., to assess the accuracy of the model) [47]. We then set Maxent to generate 10,000 background points at random from the study space for each taxon (see details of models in S1 Table).

To test the accuracy of the models, we used the Area Under the Curve (AUC) of the Receiver Operating Characteristic (ROC) plot. When absence data are not available, AUC scores represent the ability of the model to distinguish presence from background data [38]. The AUC can range from 0 to 1.0; a value of 0.5 can be interpreted as random predictions, and values above 0.5 indicate a performance better than random [47]. Additionally, we performed the partial-ROC analysis [48] which considers the portion of the ROC curve that lies within the predictive range of the modeling algorithm and within the range of acceptable models in terms of an omission error beforehand (i.e., these results are expressed as ratios; [49]). We calculated these ratios in R [50] using ENMGadgets [51]. Values of AUC ratios depart from unity as the model’s ROC curve improves with respect to random expectations, and this is performed by means of bootstrapping [49].

To test the hypothesis that the ecological niche in manakin species is conserved across evolutionary time, we considered the following: if allopatric sister species are nearly identical in environmental space, then the ecological niche is fairly conserved and ecological divergence (in relation to the parameters examined) has not been a major factor in speciation [5]. If, alternatively, allopatric sister species segregate in environmental space, it would suggest low niche conservatism and that ecologically-mediated selection may have had a role in speciation. We follow two approaches: (1) we use the record locations of all other taxa (*C*. *caudata*, *C*. *lanceolata*, *C*. *linearis*, *C*. *p*. *napensis*, *C*. *p*. *regina*, *C*. *p*. *pareola*, *A*. *galeata* and *A*. *bokermanni*) as independent data (testing data) for evaluating the SDMs of *C*. *boliviana*; and (2) we use *C*. *boliviana*’s occurrence locations as test data for evaluating the models of the other taxa. High AUC values would suggest conservatism of climatic tolerances; low AUC values would suggest low niche conservatism, perhaps suggesting that climatic factors were more important for species’ divergence.

### Comparison of niches

We calculated niche overlap, niche equivalency and niche similarity between taxa, following Broennimann et al. [26]; analyses were performed in R [50]. This framework quantifies niche overlap between two species, or any taxonomic, geographical or temporal groups of occurrences (called taxa or entities). It is important to highlight that these analyses only use the species’ occurrence locations and spatial climatic data to characterize the ecological niche [26], whereas Maxent uses presence records to predict probable occurrence locations over a landscape.

According to Broennimann et al. [26], the environmental space is defined by the first two axes of a PCA; it is divided into a grid of r x r cells (i.e., we set this resolution r to 100) in which each cell corresponds to a unique vector of environmental conditions present at one or more sites in geographical space. A kernel density function is used to determine a smoothed density of occurrences in each cell [26]. Niche overlap is calculated by the D metric [52,53]; it varies between 0 and 1, where 0 means no overlap and 1 complete niche overlap [26].

The niche equivalency test determines whether the niche overlap is constant when randomly reallocating the occurrences of both species between their two ranges. On the other hand, the niche similarity test addresses whether the environmental niche occupied by one taxon is more similar to the one occupied by another than expected by chance (see [26] and references within). In summary, the equivalency test asks whether two niches are identical, it randomly pools the occurrences of both species and reallocates them many times while calculating the D metric, whereas the similarity test asks whether one species’ niche model predicts the occurrence of the other [54].

### Niche overlap and phylogeny

Niche conservatism predicts an increase in climatic niche differentiation (i.e., lower niche overlap) between species with increasing phylogenetic distance [55]. Therefore, we tested whether pairwise niche differences correlated with phylogenetic distance; we did this by correlating the matrix of niche overlap values with a matrix of patristic distances. The patristic distance is defined as the sum of the lengths of the branches that link two taxa in an evolutionary tree [56]; it is based on the inferred number of substitutions per site. We obtained patristic distances from: a) a time-calibrated tree from Silva et al. [25], and b) the concatenated tree from Leite et al. (in revision) in which the branches were made ultrametric using non-parametric rate smoothing (hereafter referred as Silva and Leite phylogenies). Both trees were kindly shared by the authors.

The phylogeny by Silva et al. [25] included 11 taxa (i.e., all 9 taxa we considered plus *C*. *p*. *atlantica* and two varieties of *C*. *p*. *pareola*, *C*. *p*. *pareola N* and *C*. *p*. *pareola S*). For the purpose of our study, we only used data from the 9 taxa that we considered for our niche analyses, and corresponding to *C*. *p*. *pareola* we used *C*. *p*. *pareola S*. The phylogeny by Leite et al. included all species but no subspecies; therefore, in order for the two matrices to have the same number of taxa, we used 3 niche overlap matrices, each with data of each subspecies (i.e., *C*. *p*. *napensis*, *C*. *p*. *regina* and *C*. *p*. *pareola*). The correlation between each matrix of patristic distances and the niche overlap matrix was examined with a Mantel test. Additionally, we graphed the relationship between matrices, first with scatter plots and second, by drawing separate trees using the pairwise niche overlap distances to estimate branch lengths for each of the tree topologies (i.e., the Silva and the Leite phylogenies) using unweighted least squares and constraining branch lengths to be non-negative. In essence, if the difference in niches between two species was changing at a “neutral” rate, then the pairwise niche distance would be proportional to the genetic distance; if instead we got very different branch lengths from the phylogenies, it would suggest that pairwise niche distances are not changing by drift but due to other processes.

## Results

### Species Distribution Models (SDMs)

**Fig 2:**
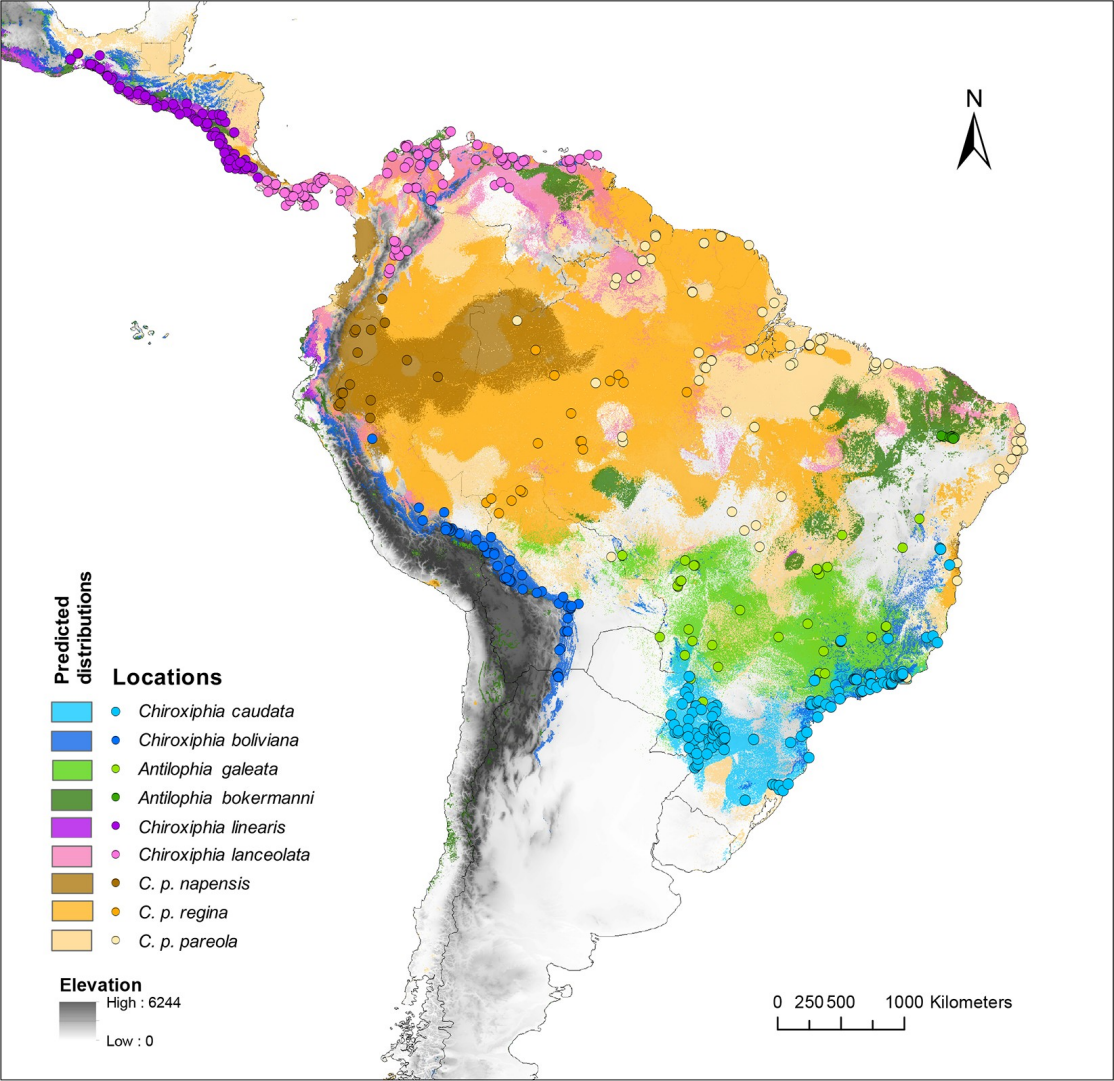
Occurrence locations and projected distributions for *Chiroxiphia* and *Antilophia* manakins. (*C*. *boliviana*, n = 66 presence records; *C*. *caudata*, n = 146; *C*. *lanceolata*, n = 93, *C*. *linearis*, n = 81, *C*. *pareola napensis*, n = 16; *C*. *pareola regina*, n = 17; *C*. *pareola pareola*, n = 74, *Antilophia galeata*, n = 40, *A*. *bokermanni*, n = 9). The shaded areas constitute binary projected distributions (suitable vs. unsuitable) based on a 10th-percentile threshold (see Methods, and S1 Fig for continuous suitability distributions). This map was made in ArcGIS 10.7 using the resulting rasters produced by Maxent (see S1 Table for more details).

All the species distribution models had high training AUC values (> 0.90) and test AUC values (> 0.86), indicating a performance much better than random (S1 Table). Mean AUC ratios were higher than 1.8 for most taxa but they were lower for the subspecies of *C*. *pareola* (range: 1.55 to 1.69) (S3 Table). The binary projected distributions for most species basically covered their known range (Fig 2, see also S1 Fig). However, for some species the predicted suitability range was much larger than their published range. For example, for *C*. *caudata* and *A*. *galeata* the models also predicted suitable areas along the eastern slope of the Andes; or for *C*. *linearis* for which the model predicted suitable areas on the western coast of Ecuador. Our models overpredicted the potential distributions of all subspecies of *C*. *pareola* as well (Fig 2, see also S1 Fig). The environmental variables that best explained each species’ distribution are listed in S3 Table.

When other taxa are used as test data for evaluating *C*. *boliviana*’s SDM, the highest test AUC values were for *C*. *caudata* and *A*. *galeata* (Table 1). High values indicate performance better than random, and our results suggest niche convergence between *C*. *boliviana*, *C*. *caudata* and *A*. *galeata*. For all subspecies of *C*. *pareola*, however, the resulting test AUC values were lower than 0.5, which indicates that training data from *C*. *boliviana* had lower ability to predict ecological niches of *C*. *pareola*’s subspecies. Consequently, there appears to be environmental niche differentiation between *C*. *boliviana* and subspecies of *C*. *pareola*. AUC values for *C*. *lanceolata*, *C*. *linearis* and *A*. *bokermanni* were a bit higher than 0.5, which also suggests performance no better than random (Table 1). Similar results were obtained when using *C*. *boliviana*’s presence records to evaluate how well other taxon’s SDMs predict *C*. *boliviana*. We found greater predictive ability as indicated by higher test AUC values between *C*. *boliviana* and *C*. *caudata* or between *C*. *boliviana* and *A*. *galeata*. Thus, these results also suggest low niche conservatism between *C*. *pareola*’s subspecies and *C*. *boliviana*. AUC values for *C*. *lanceolata*, *C*. *linearis* and *A*. *bokermanni* were higher than 0.5, but not by much, suggesting performance not better than random (Table 1).

**Table 1:** Test AUC values and AUC ratios (partial AUC) from species distribution models using two approaches: (I) Using other taxa as test data for evaluating *C*. *boliviana*; and (II) Using *C*. *boliviana* as test data for evaluating other taxa (values of more than 0.7 are in bold). Refer to S3 Table for training AUC values. Manakin species: *Cbol* (*C*. *boliviana*), *Cpnap* (*C*. *pareola napensis*), *Cpreg* (*C*. *pareola regina*), *Cppar* (*C*. *pareola pareola*), *Ccau* (*C*. *caudata*), *Clan* (*C*. *lanceolata*), *Clin* (*C*. *linearis*), *Agal* (*Antilophia galeata*), and *Abok* (*A*. *bokermanni*).

	**I.** When other taxa are used as test data for evaluating *C*. *boliviana*							
	***Cpreg***	***Cppar***	***Cpnap***	***Ccau***	***Clan***	***C*. *lin***	***Agal***	***Abok***
**Test AUC**	0.47	0.36	0.44	**0.76**	0.50	0.55	**0.72**	0.54
**AUC ratio (Mean ± SD)**	1.96	1.33 ± 0.47	1.96	1.28 ± 0.04	1.04 ± 0.03	1.04 ± 0.05	1.08 ± 0.12	1.95
	**II.** When *C*. *boliviana* is used as test data for evaluating other taxa							
	***Cpreg***	***Cppar***	***Cpnap***	***Ccau***	***Clan***	***C*. *lin***	***Agal***	***Abok***
**Test AUC**	0.54	0.33	0.53	**0.79**	0.51	0.59	0.68	0.54
**AUC ratio (Mean ± SD)**	1.97	0.91 ± 0.31	1.31 ± 0.46	1.06 ± 0.04	1.97	1.99	1.30 ± 0.47	1.96

### Niche comparisons

#### *C*. *boliviana* vs. other taxa

*C*. *boliviana* had high niche overlap only with *C*. *caudata* (D = 0.62, Table 2). The hypothesis of niche equivalency between these species could not be rejected, suggesting that they occupy environments that are more equivalent than expected by chance. However, niche similarity was rejected (i.e., this test examines whether the environmental niche occupied by *C*. *caudata* is more similar to the one occupied by *C*. *boliviana* than expected by chance, and vice-versa) (Table 2). When comparing both species’ ranges in environmental space, the first component of the PCA explained 29% of the variation and represents a gradient of decreasing precipitation of coldest and driest quarter and increasing precipitation seasonality (S2 Fig, S4 Table). This makes sense given that both species inhabit ecoregions with high precipitation variability [24].

**Table 2:** Analyses of niche overlap, niche equivalency and niche similarity of *Chiroxiphia* and *Antilophia* manakins, following Broennimann et al. [26]. Niche overlap measures levels of intersection between two species’ ranges, niche equivalency measures whether the niche overlap is constant when randomly reallocating occurrences of both species between their two ranges, and niche similarity asks whether one species’ niche can predict the occurrence of the other.

Species pairwise comparison	Niche overlap Schoener’s D	Equivalency * (*P*-value)	Similarity ** (*P*-value) Sp. 1 →Sp. 2 ***	Similarity ** (*P*-value) Sp. 2 →Sp. 1 ***
*C*. *boliviana* vs. *C*. *caudata*	**0.62**	s	0.01	0.13
*C*. *boliviana* vs. *C*. *lanceolata*	0.39	ns	0.01	0.01
*C*. *boliviana* vs. *C*. *linearis*	0.42	ns	0.01	0.01
*C*. *boliviana* vs. *A*. *galeata*	0.27	ns	<0.01	<0.01
*C*. *boliviana* vs. *A bokermanni*	0.01	ns	0.01	0.01
**Comparison between *C*. *boliviana* and *C*. *pareola*’s subspecies**				
*C*. *boliviana* vs. *C*. *p*. *pareola*	0.39	ns	0.01	0.01
*C*. *boliviana* vs. *C*. *p*. *napensis*	0.07	ns	0.37	0.01
*C*. *boliviana* vs. *C*. *p*. *regina*	0.18	ns	0.04	0.01
**Comparison among *C*. *pareola*’s subspecies**				
*C*. *p*. *pareola* vs. *C*. *p*. *regina*	0.38	ns	0.29	0.01
*C*. *p*. *pareola* vs. *C*. *p*. *napensis*	0.18	ns	0.01	0.03
*C*. *p*. *napensis* vs. *C*. *p*. *regina*	0.23	ns	0.01	0.21
**Other comparisons**				
*A*. *galeata* vs. *A*. *bokermanni*	0.02	ns	0.07	0.01
*C*. *lanceolata* vs. *C*. *linearis*	**0.65**	s	0.01	0.01
*A*. *galeata* vs. *C*. *caudata*	0.35	ns	0.01	0.05
*C*. *lanceolata* vs. *C*. *p*. *napensis*	0.12	ns	0.06	0.01
*C*. *lanceolata* vs. *C*. *p*. *pareola*	**0.77**	s	0.01	0.01
*C*. *lanceolata* vs. *C*. *p*. *regina*	0.45	ns	0.23	0.01
*C*. *p*. *pareola* vs. *C*. *caudata*	0.23	ns	0.01	0.01
*C*. *p*. *pareola* vs. *A*. *galeata*	0.33	ns	0.03	0.01

* Equivalency: ns = non-significant, the hypothesis of niche equivalency is rejected; s = significant, niche equivalency cannot be rejected.

** Similarity: if *P* < 0.05, hypothesis of niche similarity is rejected; if *P* > 0.05, niche similarity cannot be rejected.

*** Arrows mean the direction to which the range of one species is overlaid on the other range.

The ecological niche of *C*. *boliviana* in comparison with *C*. *pareola*’s subspecies showed low niche overlap, and the hypotheses of niche equivalency and niche similarity were rejected (Table 2). The first component explained a large portion of the variation in all three comparisons (30–45% of the variation; S4 Table, S2 Fig). In the comparison with *C*. *p*. *regina* and *C*. *p*. *napensis*, the first axis was defined by decreasing annual precipitation and increasing precipitation seasonality; precipitation of the driest quarter was also important in *C*. *p*. *napensis*’ case, and mean temperature of the driest quarter in *C*. *p*. *regina*’s case (S2 Fig, S4 Table). In the comparison with *C*. *p*. *pareola*, the first axis represented a gradient with decreasing precipitation of coldest quarter and mean temperature of driest quarter, and increasing temperature seasonality (S4 Table, S2 Fig). In all three comparisons, the second axis explained between 12 and 17.5% of the variation and the contribution of environmental variables was diverse (S4 Table, S2 Fig).

In comparisons with other taxa, *C*. *boliviana* had marginally moderate niche overlap with *C*. *linearis* and *C*. *lanceolata* (D = 0.42 and D = 0.39, respectively; Table 2). However, the hypotheses of niche equivalency and similarity were rejected. The PCA that described the environmental niche of *C*. *boliviana* and *C*. *linearis* explained 46.8% of the variation; it was defined by increasing precipitation seasonality and mean temperature of the driest quarter, and increasing overall maximum NDVI, in its first component (S2 Fig, S4 Table). The second component described a gradient of decreasing annual precipitation and that of the coldest quarter, and increasing temperature seasonality (S2 Fig, S4 Table).

In the rest of comparisons (Table 2), two cases stand out: 1) *C*. *lanceolata* vs. *C*. *linearis* and 2) *C*. *lanceolata* vs. *C*. *p*. *pareola*, because they had high values of niche overlap (D = 0.65 and D = 0.77, respectively) and the hypotheses of niche equivalency could not be rejected. Species in the first comparison inhabit both dry and humid lowland forests, and their life histories share similarities [24]. Species in the second comparison were distributed in all types of lowland forests in the north of South America (Fig 2, S1 Fig).

### Niche overlap and phylogeny

**Fig 3:**
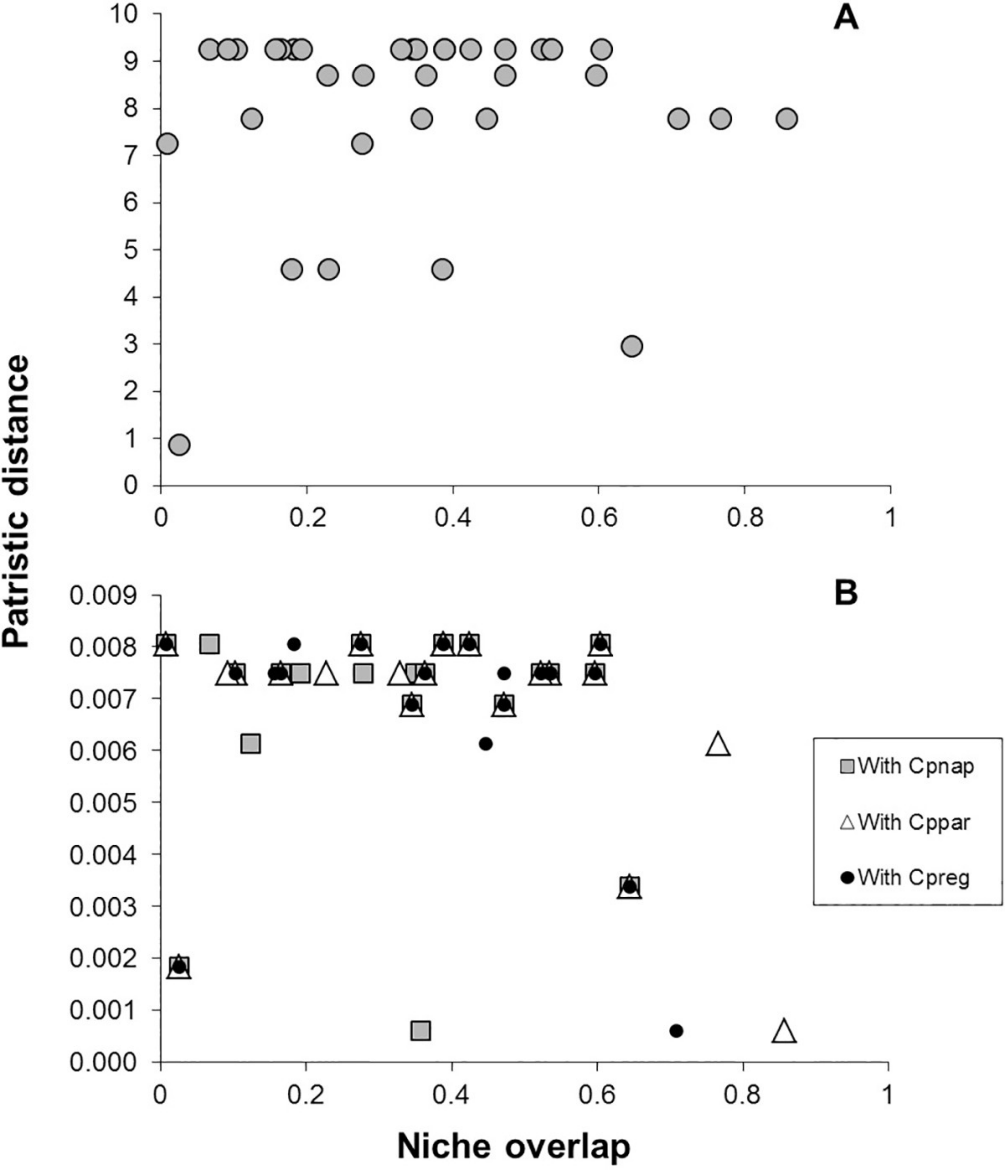
**Scatter plots showing the relationship between niche overlap (x axis) and patristic distance (y axis).** Patristic distances were calculated from time-calibrated trees obtained from the Silva phylogeny (A) and the Leite phylogeny (B).

Mantel tests showed that there was no significant relationship between niche differentiation and phylogenetic distance, neither in the case of the Silva phylogeny (Mantel *r* = 0.084, *P* = 0.25), nor the Leite phylogeny (niche overlap with *C*. *p*. *napensis*: Mantel *r* = -0.126, *P* = 0.73; with *C*. *p*. *regina*: Mantel *r* = -0.153, *P* = 0.80; with *C*. *p*. *pareola*: Mantel *r* = -0.167, *P* = 0.79) (Fig 3). Similarly, the least-squares trees showed that the degree of niche overlap resulted in branch lengths that were not consistent with the phylogeny (S3 Fig), suggesting that niche distances were not changing by drift but rather due to other processes.

## Discussion

Through the use of varied approaches, our study revealed that niche divergence may have been a major process in the diversification of taxa in this clade of manakins. It confirmed some results reported by Anciães and Peterson [19,29] that *C*. *boliviana* occurs at montane humid forests at higher elevations than other manakins, and that its ecological niche differs significantly from that of *C*. *pareola*, its sister species according to the phylogeny they used [19]. However, we give further insights into the differentiation of *C*. *boliviana*’s ecological niche. The three approaches we followed showed first that the environmental conditions in which *C*. *boliviana* is distributed are comparable to those of *C*. *caudata* and *A*. *galeata*; second, that especially between *C*. *boliviana* and *C*. *caudata* there was a high niche equivalency, and level of intersection between their ranges (i.e., niche overlap); and thirdly, that the ecological niches in this clade formed by *Chiroxiphia* and *Antilophia* segregated more than expected given their phylogenetic distances, suggesting niche divergence rather than niche conservatism. We propose that while allopatric speciation may have been important for the speciation of *C*. *boliviana*, ecologically mediated selection could not be ruled out as a factor in its speciation. Ecological selection occurs when new environmental conditions appear, including geographic heterogeneity; this ecologically-based divergent selection can create genetic diversification from the original population and therefore speciation [9,57,58]. Further, we propose that ecological selection may have also had an important role in speciation for the other *Chiroxiphia* and *Antilophia* manakins.

Here, our main objective was to compare *C*. *boliviana*’s niche with other South American manakin taxa at a landscape, coarse spatial resolution to allow a comparison among species with very different geographic range sizes. Given that *C*. *boliviana* is the species that reaches the highest elevations in its genus, we wanted to get some insights on environmental conditions that characterize its ecological niche. We believe these types of comparisons are very useful in general, because under climate change, historical envelopes are expected to shift upslope and species distributions are expected to follow; this could have significant effects on avian communities [59].

Most of the models developed here over-predicted the geographic distribution of species. The model of *C*. *boliviana*, for example, predicted a distribution on its known historic range along the eastern slope of the Andes primarily, but it also predicted suitable areas in the southeast of Brazil, in the Atlantic forest. The models for *C*. *caudata* and *A*. *galeata*, predicted their known ranges as well, but also suitable areas along the eastern slope of the Andes, particularly for *C*. *caudata*. These results highlight the resemblance in the ecological niches of *C*. *boliviana* and *C*. *caudata* (see below). Furthermore, the geographic distribution was largely over-predicted for all subspecies of *C*. *pareola*, perhaps as a consequence of modelling species with such wide distributions [60]. Over-prediction in our models might also reflect ecological differentiation of these taxa in dimensions that we did not examine [5]. Climate variables describe the fundamental niche and therefore act at large scales, whereas other aspects of the ecological niche of a species (e.g. vegetation, distribution of nesting or food resources, distribution of leks; [61,62]) and divergent selection pressures are manifested at much finer spatial scales than climatic variation. Important aspects describing the realized niche of a species, such as biotic interactions, are overlooked when distributions are modeled at such large geographic scales [7,17], although they can be very important in determining distributions. Freeman [63] for example, studying sister species pairs of tropical montane birds shows that competitive interactions upon secondary contact are a common mechanism driving elevational divergence. We did not consider accessible areas over relevant time periods when selecting the geographic extent for model calibration, which can result in an overestimation of niche conditions [64].

Several studies on a broad range of organisms have examined the ability of ecological niche models to reveal information about niche evolution and differentiation [5–6,65–71]. Using similar methodology as ours, many of these show niche conservatism in evolutionary time between sister taxon pairs (e.g., in birds [70,71], in birds, mammals and butterflies [65], in salamanders [6], in plants [68]), though others show niche divergence (e.g., in birds [66], in lizards [67]). Niches of *C*. *caudata* and *C*. *boliviana* showed important resemblances in environmental space and demonstrated high niche overlap and equivalency; each species could predict the distribution of the other species to a reasonable degree. Both species inhabit topographically diverse areas, with great environmental heterogeneity (this study and [19]). *C*. *caudata* is found in the understory of the southern coastal Atlantic Forest in Brazil, occurring in lowland and montane evergreen forests as well as secondary forests [24,72]. Comparatively, *C*. *boliviana* inhabits semi-deciduous to humid montane/hill forests at 600–2600 m.a.s.l., where it is found both in forest interior and at edges of primary and secondary forests along the eastern slope of the southern Andes [24,73]. These results show that even though *C*. *boliviana* and *C*. *caudata* occur in areas with different climate characteristics, their respective ranges were more similar than expected by chance. Likewise, Rice et al. [66] examined similar questions between pairs of *Aphelocoma* jays and found low predictability and low niche similarity between closely related species; Zink [14] explored the role of niche conservatism and divergence in shaping species ranges and found a lack of niche divergence between sister species of aridland birds; Jiguet et al. [74] studied two sister species of cotingas and found that even with their similar niches, one species could not predict the other. Studies comparing niches between subspecies find both niche similarities (e.g., between eastern and western subspecies of *Passerina ciris* in North America; [70]) and divergences (e.g., subspecies of the woodpecker *Colaptes auratus* and the warbler *Setophaga coronata*; [75]). Analogous questions assessing whether ecological niches between sister species can be predicted over space and time, were explored with plants [68,76].

Phylogenetic niche conservatism (PNC) refers to the tendency for lineages to retain ancestral ecological characteristics over time; however, Pyron et al. [58] argue that if populations are experiencing rapid ecological change, selection for their current niche (PNC) may actually result in niche divergence. They proposed a theoretical framework in which they discuss the mechanisms by which PNC can act as a fundamental driving force in speciation [58]. This process can lead to three potential patterns: a) niche constraints (speciation occurs by internal mechanisms), b) niche conservatism (similarity of ecological niches over evolutionary timescales), and c) niche divergence (geographic and ecological variation are large; local adaptation will lead populations to diverge from their ancestral niche as they track their instantaneous niche). Some tests for PNC have been proposed using distribution models [58,77]. If sister species pairs are less similar than expected under a null model on the phylogeny, it would be indicative of PNC due to directional ecological selection driving speciation; on the other hand, if species are more similar than expected under their phylogenetic relationships, it would be indicative of PNC due to stabilizing selection. This is essentially what we tested for by comparing the ecological niches of species’ pairs in light of their phylogenetic distances and relationships. The phylogeny proposed by Anciães and Peterson [19], reported *C*. *boliviana* as sister species to *C*. *pareola*. We found that these two species (with either or all the subspecies) segregated in environmental space more than expected given their phylogenetic distance, suggesting that perhaps ecologically mediated selection may have had an important role in speciation. If we consider *C*. *boliviana* as sister to *Antilophia*, as the Silva phylogeny denotes, these taxa also segregated in environmental space more than expected given their phylogenetic distance (the Leite phylogeny suggests *C*. *boliviana* is sister to all other ingroup species, making it harder to directly compare). We did not find a significant relationship between niche overlap and phylogenetic distance (regardless of which phylogeny we used), though the slight negative trend might suggest ecological selection and an increase of ecological differences over evolutionary time, a pattern more consistent with niche divergence.

Previous research has examined the phylogenetic relationships between sister species of vertebrates (i.e., birds, mammals) in ecoregions of South America that are geographically separated, and many have found interesting taxonomic affinities between regions, which could explain the niche similarities that we found between *C*. *boliviana* and *C*. *caudata*. These studies include comparisons between the Itatiaia highlands of southeastern Brazil and the Bolivian Andes (e.g. [78]), between the Amazon and Atlantic forests (e.g. [9,79–81]), between the tropical Andes and the Amazon (e.g. [82]), or between seasonally dry tropical forests (SDTFs) (e.g. Caatinga, interandean valleys in Ecuador, Peru and Bolivia; [83]). For instance Sick [78], suggested that there was a band of continuous vegetation extending between the Andes and southeastern Brazil, which served as a colonization corridor for many plant and bird species (e.g. *Scytalopus novacapitalis*, *Caprimulgus longirostris*, *Schizoeaca moreirae*, etc.). Combining phylogenetic with distributional data, Batalha-Filho et al. [81] examined taxa of New World suboscines with disjunct Amazonian/Atlantic forest distributions with the objective of depicting historical connections between these biomes. They report that the Atlantic and Amazonian forests were connected in the past and they hypothesize different pathways for the dispersal of organisms. Their study considered three *Chiroxiphia* species (i.e., *C*. *boliviana*, *C*. *caudata* and *C*. *pareola*) and found that *C*. *boliviana* had a closer relationship to *C*. *caudata* than to *C*. *pareola*; they also estimated a recent time of split (4.17 mya) between *C*. *boliviana* and *C*. *caudata*. The Batalha-Filho et al. [81] study, combined with our study and the two phylogenies we used, suggest lack of agreement in placement of *C*. *boliviana* within the clade. The phylogeny by Silva et al. [25] found that *C*. *boliviana* is more closely related to *Antilophia* than to other *Chiroxiphia*; however, phenotypic and behavioral differences suggest otherwise. On the other hand, the Leite maximum likelihood phylogeny places *C*. *boliviana* sister to *Antilophia* and to other *Chiroxiphia* species (though there was some uncertainty about this relationship).

Overall, this study has given us insights on the ecological niche of *C*. *boliviana* in comparison to other closely related manakins. It has been able to depict some relevant ecological niche differences and similarities among manakin taxa, and set up the potential for further examining niche divergence in relation to morphological and molecular divergence.

## Supporting information

S1 Fig Continuous projections of the distribution models for each manakin taxon considered: *Chiroxiphia boliviana*, *C. caudata*, *C. lanceolata*, *C. linearis*, *C. pareola napensis*, *C. pareola pareola*, *C. pareola regina*, *Antilophia galeata*, and *A. bokermanni*. These maps were made in ArcGIS 10.7 using the resulting rasters produced by Maxent.
(TIF)

S2 Fig Ecological niches in environmental space; each row of panels represents a pairwise comparison between *C. boliviana* and one other taxa. (A) comparisons with *C*. *caudata*, *C*. *lanceolata* and *C*. *linearis*. (B) comparisons with subspecies of *C*. *pareola*. (C) comparisons with *A*. *galeata* and *A*. *bokermanni*. Environmental space is represented by 13 environmental variables, reduced to two dimensions by a principal component analysis; contributions of environmental variables on the axes of the PCA are given in S2 Table. Grey shading shows the density of occurrences of the species by cell; the solid line illustrates 100% and the dashed line the 50% of the available (background) environment, following Broenniman et al. [26].
(TIF)

S3 Fig Least-squares trees with the (A) Silva phylogeny and the (B) Leite phylogeny (with Cpnap, Cppar and Cpreg).
(TIF)

S1 Table Checklist of the species distribution modeling. We followed the guidelines provided by Feng et al. [27].
(DOCX)

S2 Table Correlation matrix among the 23 environmental variables considered initially (correlation coefficients > 0.8 are in bold). The 13 variables used for the species distribution models are painted in grey cells.
(DOCX)

S3 Table Contributions (percentage of total) of environmental variables (the highest values are in bold) and AUC values for each distribution model developed. In this analysis, we used 75% of the occurrence points for training and 25% for testing the models. Environmental variables: annualpp (annual precipitation), ppcoldqua (precipitation of coldest quarter), ppdryqua (precipitation of driest quarter), ppseason (precipitation seasonality), ppwarmqua (precipitation of the warmest quarter), maxtwarmmo (maximum temperature of warmest month), meantdryqua (mean temperature of driest quarter),), ppcoldqua (precipitation of coldest quarter), ppdryqua (precipitation of driest quarter), ppwarmqua (precipitation of warmest quarter), ppseason (precipitation seasonality: standard deviation *100), tseasoncv (temperature seasonality), overall maximum NDVI (maxndvi), coefficient of variation NDVI (cvndvi), eastness, northness and slope. Manakin species: *Cbol* (*C*. *boliviana*), *Cpnap* (*C*. *pareola napensis*), *Cpreg* (*C*. *pareola regina*), *Cppar* (*C*. *pareola pareola*), *Ccau* (*C*. *caudata*), *Clan* (*C*. *lanceolata*), *Clin* (*C*. *linearis*), *Agal* (*Antilophia galeata*), and *Abok* (*A*. *bokermanni*).
(DOCX)

S4 Table Pairwise niche comparisons in multivariate space: Factor loadings of environmental variables (the 3 variables that contribute the most are in bold) and explained variation by the principal component axes. See S2 Fig for comparisons.
(DOCX)
