# Supplementary figures and images for: Ecological niche differentiation in Chiroxiphia and Antilophia manakins (Aves: Pipridae)
Source: PLoS One. 2021 Jan 13;16(1):e0243760. doi: 10.1371/journal.pone.0243760 (PMC7806125; doi:10.1371/journal.pone.0243760)

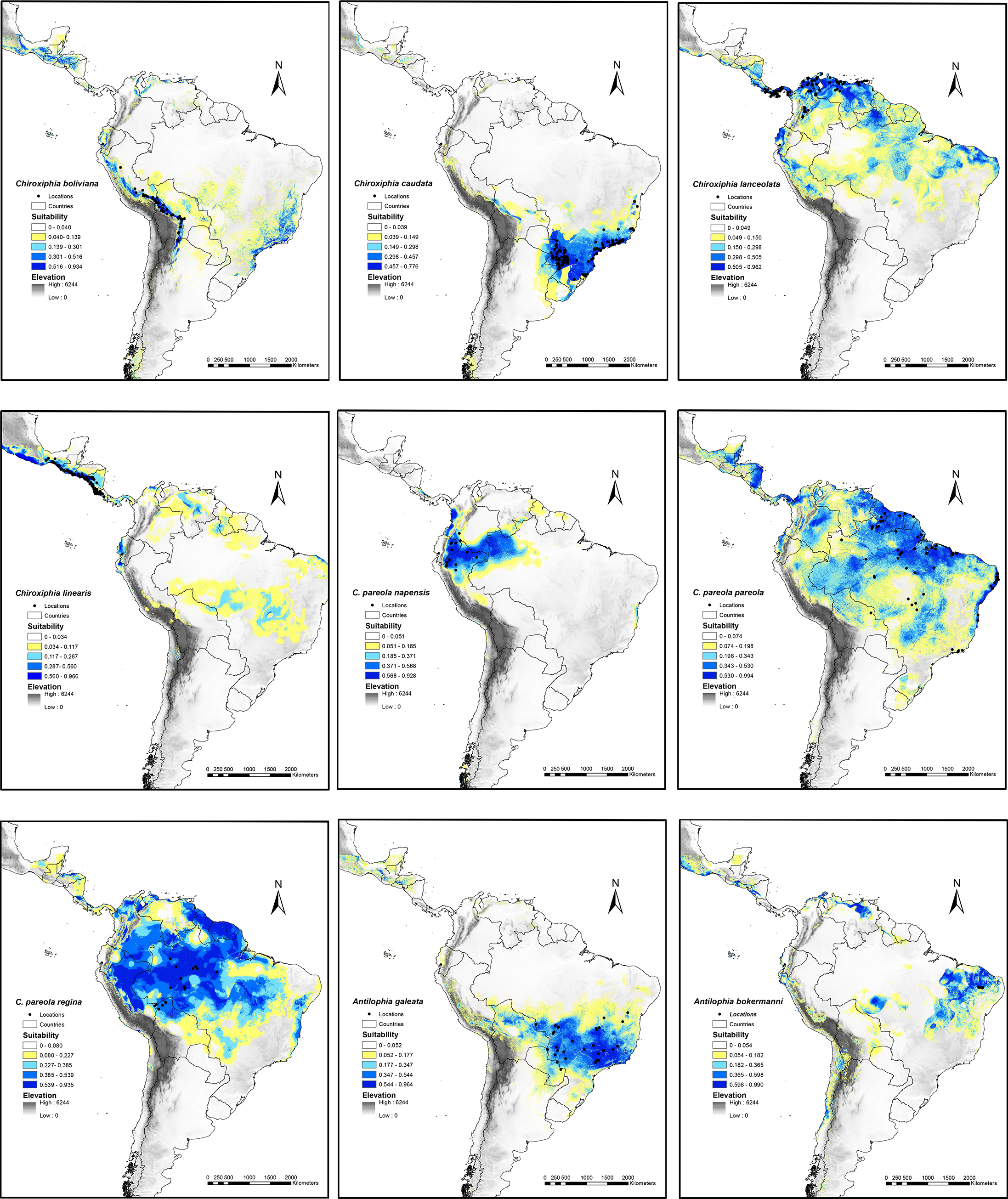

Supplement: S1 Fig — These maps were made in ArcGIS 10.7 using the resulting rasters produced by Maxent. (TIF) [file pone.0243760.s001.tif]

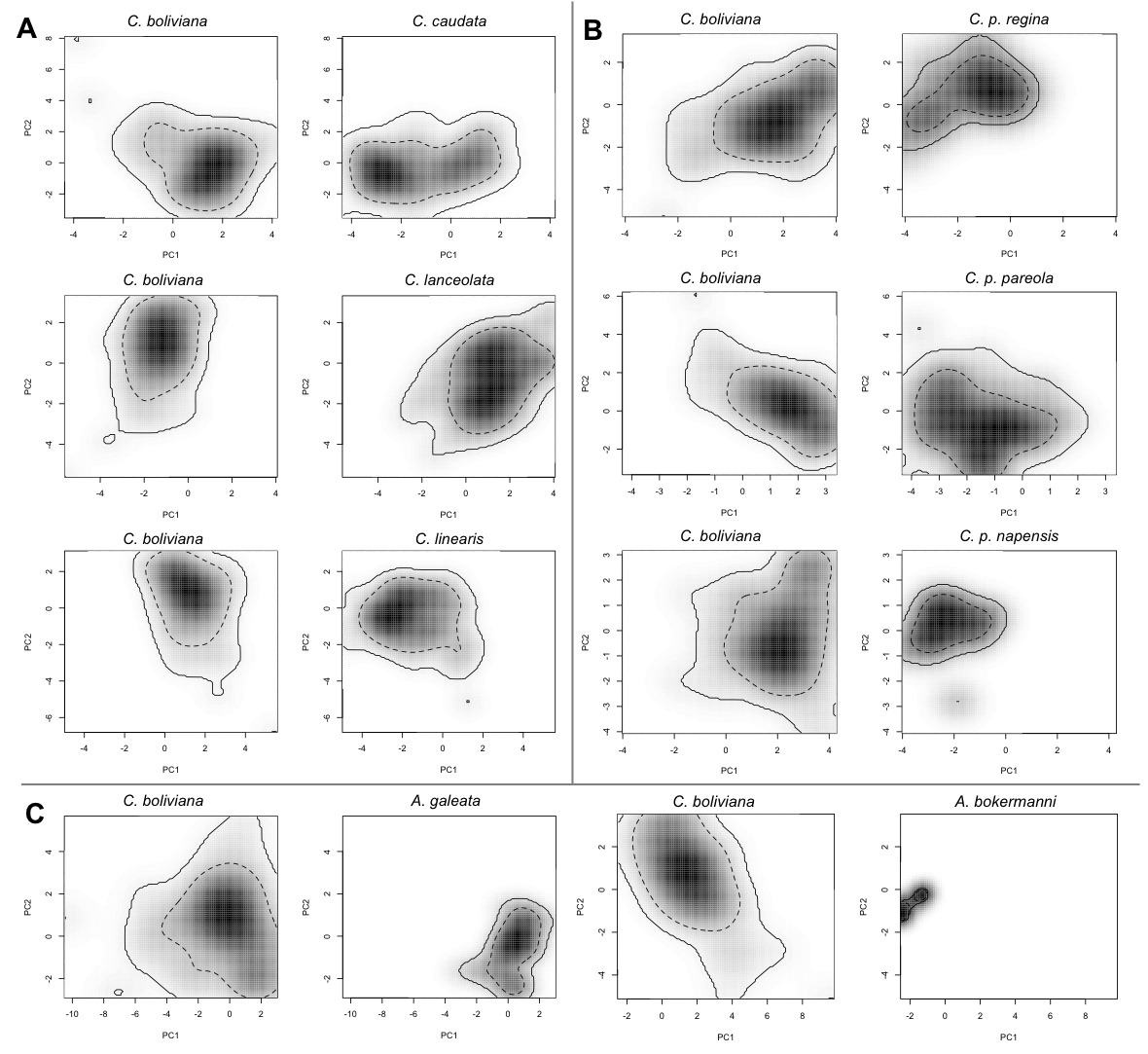

Supplement: S2 Fig — (A) comparisons with C. caudata, C. lanceolata and C. linearis. (B) comparisons with subspecies of C. pareola. (C) comparisons with A. galeata and A. bokermanni. Environmental space is represented by 13 environmental variables, reduced to two dimensions by a principal component analysis; contributions of environmental variables on the axes of the PCA are given in S2 Table. Grey shading shows the density of occurrences of the species by cell; the solid line illustrates 100% and the dashed line the 50% of the available (background) environment, following Broenniman et al. [26]. (TIF) [file pone.0243760.s002.tif]

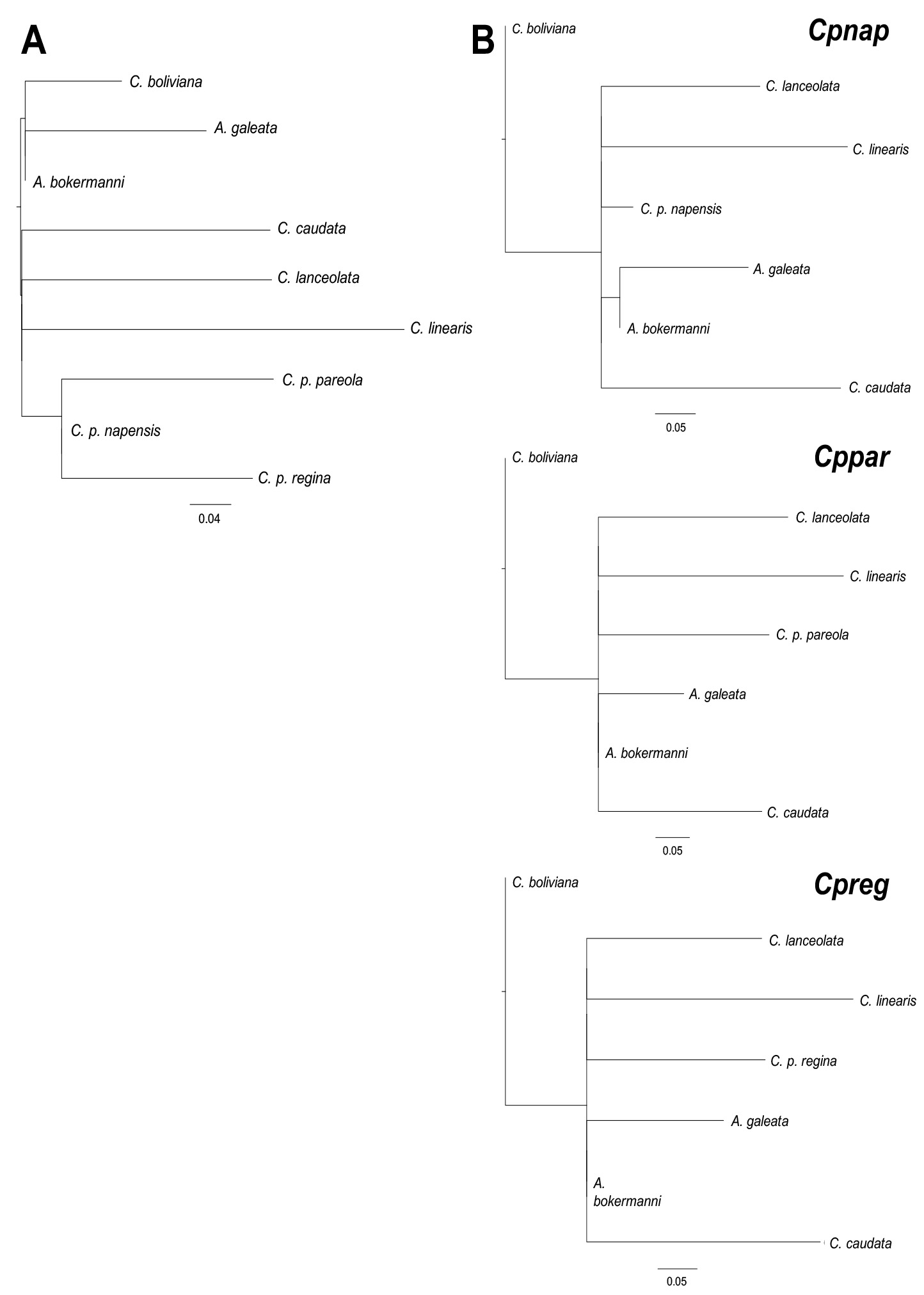

Supplement: S3 Fig — Least-squares trees with the (A) Silva phylogeny and the (B) Leite phylogeny (with Cpnap, Cppar and Cpreg). (TIF) [file pone.0243760.s003.tif]
