# Supplementary material for: Ecological niche differentiation in Chiroxiphia and Antilophia manakins (Aves: Pipridae)
Source: PLoS One. 2021 Jan 13;16(1):e0243760. doi: 10.1371/journal.pone.0243760 (PMC7806125; doi:10.1371/journal.pone.0243760)
Supplement: S1 Table — We followed the guidelines provided by Feng et al. [27]. (DOCX) [file pone.0243760.s004.docx]

**S1 Table. Checklist of the species distribution modeling.** We followed the guidelines provided by Feng et al. [1]**.**

| Workflow | Category | What to report | Your method | Your rationale |
| --- | --- | --- | --- | --- |
| (A) Obtaining and processing occurrence data | metadata | (A1) source of occurrence data | Personal sources observations, records provided by M. Anciães (for *A. galeata*, *C. caudata*, *C. lanceolata* and *C. linearis*), I. Areta (several records for *C. boliviana* in southern Bolivia and northern Argentina), J. P. Gomez (several records for *C. lanceolata* in Colombia), some records from the CBF (Colección Boliviana de Fauna), and records from citizen science and natural history museums available on the internet through e-bird, GBIF and ORNIS. | We were interested in extending the study by Anciães & Peterson [2] by using some of their same occurrence records, but going further and complementing this dataset. |
|  |  | (A2) download date; version of data source | Occurrences were provided by M. Anciães in 2014. Occurrences were downloaded from ORNIS and e-bird in December 2015 and in January 2016 from GBIF. Records by I. Areta and J.P. Gomez were provided in January 2016. |  |
|  |  | (A3) basis of records | The records provided by M. Anciães, those from CBF, ORNIS and GBIF included mostly museum specimens. My records, those from e-bird, and those provided by I. Areta and J.P. Gomez were mainly observations. |  |
|  |  | (A4) spatial extent | We considered the spatial extent of the Neotropics, from the south of Mexico to the north of Argentina and south of Brazil, which encompasses the distribution range of *Chiroxiphia* and *Antilophia*. | We concentrated on the continental South and Central America (that is why we did not include the subspecies *C. pareola atlantica*, also because it’s an isolated population; [3]). Our main focus for study was *Chiroxiphia boliviana* in comparison with other *Chiroxiphia* and *Antilophia* manakins. Further, *C. p. atlantica* |
|  |  | (A5) temporal range | Records encompass a range of 145 years, from 1871 to 2016. |  |
|  | processing | (A6-1) duplicate coordinates | Location data were first mapped to inspect for georeferencing errors and to avoid duplication. |  |
|  |  | (A6-2) spatial/environmental outlier; error | Location data were first mapped to inspect for georeferencing errors; we discarded obvious misplaced localities. |  |
|  |  | (A6-3) spatial/coordinate uncertainty |  |  |
|  |  | (A7-1) sampling bias | We tried to use only records that were at least 1 km apart, to reduce sampling bias, however, in few cases we used record locations that were closer because we wanted to have a complete representation of each species’ range. |  |
|  |  | (A7-2) spatial autocorrelation |  |  |
| (B) Obtaining and processing environmental data | metadata & processing | (B1) source | We used 8 climate variables downloaded from WorldClim [4]; 3 variables that describe topography, obtained from DIVA-GIS [5]; and 2 variables that described vegetation (NDVI - [Normalized Difference Vegetation Index]) downloaded from the Copernicus Global Land Service program (available at http://land.copernicus.vgt.vito.be/PDF/portal/Application.html#Home). |  |
|  |  | (B2) download date; version of data source | All the variables were downloaded in January 2014 (WorldClim version 1.4, [4]; DIVA-GIS version 7.1.7, [5]. |  |
|  |  | (B3) spatial resolution | All the environmental variables were in raster format and were prepared in ArcGIS 10.3 [6] to align in geographic space using a WGS84 datum system, and to match in spatial extent and cell size (~1 km^2^ cell size, or 0.00833 decimal degrees) |  |
|  |  | (B4) temporal range | The climate data from WorldClim 1.4 are for 1960-1990. For NDVI variables, we used data from 9 years, January 2005 to December 2013. |  |
| (C) Model calibration | data input | (C1) modeling domain | Locality data were first overlaid on base terrain maps in ArcGIS 10.4 [6] with a global country boundaries layer [5]. |  |
|  |  | (C2) number of background data |  |  |
|  |  | (C3) sampling method for background data | Models were performed in Maxent 3.3.3 [7] with the default settings. To develop models for each species and subspecies, we first randomly partitioned each species’ data (occurrence locations) into two data sets: 75% used as training data (i.e., to formulate the model parameters) and 25% as test data (i.e., to assess the accuracy of the model) [8,9]. We then set Maxent to generate 10,000 background points at random from the study space for each taxon. |  |
|  |  | (C4) variable selection | To reduce the number of environmental variables, we followed methods by Parra et al. [10] and plotted 1,000 random points within the geographic study area (from southern Mexico to northern Argentina), extracted the associated environmental variables and with these values performed a correlation matrix (S2 Table). We removed variables that had a coefficient of correlation > 0.8 with other environmental variables. |  |
|  | algorithm | (C5) name | Models were performed in Maxent 3.3.3 [7] which uses the principle of maximum entropy. |  |
|  |  | (C6) version of algorithm and software | Maxent 3.3.3 version 2013 [7]. |  |
|  |  | (C7) parameterization | Models were run using the default regularization values (i.e. regularization penalizes the use of too many model parameters; it forces Maxent to focus on the most important features by avoiding overfitting). |  |
| (D) Model transfer and evaluation | evaluation | (D1) evaluation index | To test the accuracy of the models, we used the Area Under the Curve (AUC) of the Receiver Operating Characteristic (ROC) plot. Additionally, we performed the partial-ROC analysis which considers the portion of the ROC curve that lies within the predictive range of the modeling algorithm and within the range of acceptable models in terms of an omission error beforehand [11,12]. We calculated these ratios in R [13] using ENMGadgets [14] |  |
|  |  | (D2) threshold for evaluation index | Given that specificity (i.e., proportion of cells correctly predicted as absence cells in relation to all absence cells) cannot be calculated with presence-only data, a threshold of predicted probability was selected. |  |
|  |  | (D3) dataset used to evaluate models | To develop models for each species and subspecies, we first randomly partitioned each species’ data (occurrence locations) into two data sets: 75% used as training data (i.e., to formulate the model parameters) and 25% as test data (i.e., to assess the accuracy of the model). |  |
|  | output | (D4) format/transformation | We used the logistic model output. The logistic model output is a transformation of the relative occurrence rate, which describes the relative probability of presence [15]; it is a continuous surface of values ranging from 0 to 1 (i.e., high values indicate a high probability of occurrence). |  |
|  |  | (D5) threshold | The resulting models were converted to presence-absence using a 10th-percentile training presence threshold (this identifies the top 90% of training samples). We used ArcGIS 10.7 [6] with the resulting rasters, with a global country boundaries layer [5] and a Digital Elevation Model raster [16] to make maps of the discrete (Fig 2) and continuous (S1 Fig) suitable distribution ranges of each species. |  |
|  | extrapolation | (D6) novelty of projected environments compared with training environments |  |  |
|  |  | (D7) collinearity shift between training and projected environments |  |  |
|  |  | (D8) extrapolation strategy |  |  |
|  | metadata | (D9) source | See (B1) |  |
|  |  | (D10) download date; version of data source | See (B2) |  |
|  |  | (D11) spatial resolution | See (B3) |  |
|  |  | (D12) temporal range | See (B4).  The ecological niche models were performed from February to April 2016. |  |
