# Supplementary material for: Ecological niche differentiation in Chiroxiphia and Antilophia manakins (Aves: Pipridae)
Source: PLoS One. 2021 Jan 13;16(1):e0243760. doi: 10.1371/journal.pone.0243760 (PMC7806125; doi:10.1371/journal.pone.0243760)
Supplement: S2 Table — The 13 variables used for the species distribution models are painted in grey cells. (DOCX) [file pone.0243760.s005.docx]

**S2 Table. Correlation matrix among the 23 environmental variables considered initially (correlation coefficients > 0.8 are in bold).** The 13 variables used for the species distribution models are in grey cells.
