# Supplementary material for: Ecological niche differentiation in Chiroxiphia and Antilophia manakins (Aves: Pipridae)
Source: PLoS One. 2021 Jan 13;16(1):e0243760. doi: 10.1371/journal.pone.0243760 (PMC7806125; doi:10.1371/journal.pone.0243760)
Supplement: S3 Table — In this analysis, we used 75% of the occurrence points for training and 25% for testing the models. Environmental variables: annualpp (annual precipitation), ppcoldqua (precipitation of coldest quarter), ppdryqua (precipitation of driest quarter), ppseason (precipitation seasonality), ppwarmqua (precipitation of the warmest quarter), maxtwarmmo (maximum temperature of warmest month), meantdryqua (mean temperature of driest quarter),), ppcoldqua (precipitation of coldest quarter), ppdryqua (precipitation of driest quarter), ppwarmqua (precipitation of warmest quarter), ppseason (precipitation seasonality: standard deviation *100), tseasoncv (temperature seasonality), overall maximum NDVI (maxndvi), coefficient of variation NDVI (cvndvi), eastness, northness and slope. Manakin species: Cbol (C. boliviana), Cpnap (C. pareola napensis), Cpreg (C. pareola regina), Cppar (C. pareola pareola), Ccau (C. caudata), Clan (C. lanceolata), Clin (C. linearis), Agal (Antilophia galeata), and Abok (A. bokermanni). (DOCX) [file pone.0243760.s006.docx]

**S3 Table. Contributions of environmental variables (percentage of total; the highest values are in bold) and AUC values for each distribution model developed.** We used 75% of the occurrence points for training and 25% for testing the models. Environmental variables: annualpp (annual precipitation), ppcoldqua (precipitation of coldest quarter), ppdryqua (precipitation of driest quarter), ppseason (precipitation seasonality), ppwarmqua (precipitation of the warmest quarter), maxtwarmmo (maximum temperature of warmest month), meantdryqua (mean temperature of driest quarter), ppcoldqua (precipitation of coldest quarter), ppdryqua (precipitation of driest quarter), ppwarmqua (precipitation of warmest quarter), ppseason (precipitation seasonality: standard deviation *100), tseasoncv (temperature seasonality), overall maximum NDVI (maxndvi), coefficient of variation NDVI (cvndvi), eastness, northness and slope. Manakin species: *Cbol* (*Chiroxiphia boliviana*), *Cpnap* (*C. pareola napensis*), *Cpreg* (*C. pareola regina*), *Cppar* (*C. pareola pareola*), *Ccau* (*C. caudata*), *Clan* (*C. lanceolata*), *Clin* (*C. linearis*), *Agal* (*Antilophia galeata*), and *Abok* (*A. bokermanni*).

|  | **Variable contribution per species model** | | | | | | | | |
| --- | --- | --- | --- | --- | --- | --- | --- | --- | --- |
| **Variable** | ***Cbol*** | ***Cpreg*** | ***Cppar*** | ***Cpnap*** | ***Ccau*** | ***Clan*** | ***Clin*** | ***Agal*** | ***Abok*** |
| annualpp | 0.28 | 0.67 | 11.74 | 0 | 0.36 | 9.52 | **17.59** | 12.04 | 0.73 |
| ppcoldqua | 3.62 | 2.75 | 7.70 | 2.43 | 1.94 | 3.82 | 1.58 | 7.50 | **21.87** |
| ppdryqua | 0.10 | 0.46 | 5.45 | **58.63** | 12.21 | 6.21 | 0.16 | 0.02 | 0.00 |
| ppseason | 7.03 | 20.55 | 0.18 | **20.51** | 0.79 | 2.73 | **63.88** | 0.92 | 5.90 |
| ppwarmqua | **18.85** | 2.71 | 2.00 | 0.82 | **28.61** | 4.26 | 3.53 | 21.08 | **40.56** |
| maxtwarmmo | 0.00 | 4.43 | 3.63 | 0.00 | 0.33 | 0.32 | 0.66 | 1.62 | 0.00 |
| meantdryqua | 3.41 | **25.52** | **29.36** | 0.17 | **32.50** | **13.55** | 0.55 | 3.78 | 0.00 |
| tseasoncv | 9.27 | **41.85** | 5.44 | 13.54 | 15.60 | **46.02** | 2.93 | **22.95** | 17.91 |
| maxndvi | 5.84 | 0.77 | 0.44 | 1.49 | 1.23 | 1.03 | 0.79 | **22.46** | 8.41 |
| cvndvi | 1.96 | 0.00 | **26.42** | 2.13 | 5.06 | 0.03 | 2.15 | 0.21 | 4.56 |
| eastness | 0.33 | 0.00 | 0.57 | 0.02 | 0.19 | 0.47 | 0.07 | 0.79 | 0.06 |
| northness | 0.31 | 0.11 | 1.70 | 0.00 | 1.18 | 0.95 | 0.03 | 4.43 | 0.00 |
| slope | **49.00** | 0.17 | 5.37 | 0.26 | 0.01 | 11.09 | 6.08 | 2.20 | 0.00 |
|  | **AUC and partial AUC (ratios) values per model** | | | | | | | | |
| **AUC values** | ***Cbol*** | ***Cpreg*** | ***Cppar*** | ***Cpnap*** | ***Ccau*** | ***Clan*** | ***Clin*** | ***Agal*** | ***Abok*** |
| Training AUC | 0.96 | 0.90 | 0.90 | 0.98 | 0.94 | 0.95 | 0.97 | 0.97 | 0.98 |
| Test AUC | 0.95 | 0.90 | 0.86 | 0.95 | 0.92 | 0.93 | 0.95 | 0.93 | 0.99 |
| AUC ratio (Mean ± SD) | 1.86 ± 0.05 | 1.65 ± 0.09 | 1.55 ± 0.04 | 1.69 ± 0.12 | 1.86 ± 0.03 | 1.82 ± 0.04 | 1.80 ± 0.05 | 1.77 ± 0.07 | 1.96 |

**_**
