# Supplementary material for: Ecological niche differentiation in Chiroxiphia and Antilophia manakins (Aves: Pipridae)
Source: PLoS One. 2021 Jan 13;16(1):e0243760. doi: 10.1371/journal.pone.0243760 (PMC7806125; doi:10.1371/journal.pone.0243760)
Supplement: S4 Table — See S2 Fig for comparisons. (DOCX) [file pone.0243760.s007.docx]

**S4 Table. Pairwise niche comparisons in multivariate space: Factor loadings of environmental variables (the 3 variables that contribute the most are in bold) and explained variation by the principal component axes.** See S2 Fig for comparisons.

| **Comparison** | ***Cbol* vs. *Ccau*** | | ***Cbol* vs. *Clan*** | | ***Cbol* vs. *Clin*** | | ***Cbol vs. Cpreg*** | | ***Cbol vs. Cppar*** | | ***Cbol* vs. *Cpnap*** | | ***Cbol* vs. *Agal*** | | ***Cbol* vs. *Abok*** | |
| --- | --- | --- | --- | --- | --- | --- | --- | --- | --- | --- | --- | --- | --- | --- | --- | --- |
| **Environmental variable** | **PC1** | **PC2** | **PC1** | **PC2** | **PC1** | **PC2** | **PC1** | **PC2** | **PC1** | **PC2** | **PC1** | **PC2** | **PC1** | **PC2** | **PC1** | **PC2** |
| annualpp | -5.65 | **6.55** | -2.24 | **-7.18** | -0.02 | **-7.33** | **-4.42** | -0.44 | -6.35 | 3.08 | **-4.74** | 0.27 | -5.55 | -0.78 | 3.29 | -1.38 |
| ppcoldqua | **-8.42** | 2.64 | 3.25 | **-6.71** | -1.74 | **-6.23** | -4.40 | -0.46 | **-6.99** | -1.23 | -4.73 | 0.29 | **-6.48** | -0.36 | 2.43 | **-2.74** |
| ppdryqua | **-8.72** | 2.73 | -4.59 | -6.40 | 5.68 | -5.44 | -4.29 | **-2.15** | -5.17 | **5.84** | **-4.89** | 0.03 | **-6.79** | 0.00 | **3.52** | -1.64 |
| ppseason | **7.93** | -0.07 | 4.45 | 4.01 | **-7.13** | 1.73 | **4.46** | 1.69 | 2.98 | **-6.35** | **4.75** | 0.61 | **5.37** | -0.26 | **-3.60** | 0.19 |
| ppwarmqua | -1.89 | 4.84 | **-6.44** | -2.10 | 4.76 | -2.03 | 1.04 | -2.04 | 3.52 | 4.61 | -3.38 | 0.80 | -2.96 | -2.51 | 3.43 | 0.86 |
| maxtwarmmo | -5.44 | **-4.90** | **6.38** | -2.13 | -6.78 | -1.69 | -2.79 | **3.28** | -4.87 | -3.52 | -2.68 | **3.20** | 0.06 | **-6.51** | -2.04 | -1.11 |
| mtdryqua | -2.79 | -1.71 | **7.02** | -4.26 | **-6.96** | -3.90 | **-4.42** | 2.27 | **-7.34** | -2.44 | -4.40 | 1.39 | -1.29 | **-4.99** | -2.29 | **-2.50** |
| tseasoncv | -2.79 | -1.71 | -3.04 | **5.37** | 1.61 | **4.81** | 3.96 | 0.56 | **6.06** | -1.15 | 3.65 | 2.39 | 2.59 | **-3.97** | 0.26 | **2.92** |
| cv_ndvi | 3.03 | 4.05 | 2.35 | -2.43 | 1.35 | -1.70 | -2.38 | 0.40 | -3.18 | -2.31 | -0.90 | **-3.46** | -0.69 | 3.95 | -1.71 | -2.25 |
| maxndvi | 3.30 | **5.01** | -4.59 | -2.42 | **5.74** | -1.15 | -0.49 | **-3.33** | -0.24 | **4.11** | -2.03 | -1.89 | -2.76 | 2.43 | **3.25** | 0.27 |
| Eastness | 1.85 | -1.39 | 0.67 | 0.26 | 0.90 | 1.77 | 0.81 | 1.74 | 0.68 | -1.69 | -0.68 | -0.44 | 1.19 | 1.35 | -0.51 | -0.18 |
| Northness | 0.80 | 1.80 | 0.68 | 0.16 | 2.08 | -0.92 | -0.81 | -1.14 | -0.38 | 0.83 | -0.80 | **-2.60** | -0.13 | 1.50 | -0.25 | -0.99 |
| Slope | 2.65 | 2.49 | -2.03 | 0.44 | 3.97 | 1.22 | 1.70 | -2.73 | 4.09 | 2.67 | 1.35 | -0.57 | -0.24 | 1.54 | 1.32 | 0.69 |
| **Explained variation (%)** | 29 | 15.7 | 22.8 | 22.1 | 27.7 | 19.1 | 37.4 | 14.6 | 30.7 | 17.5 | 44.9 | 12.5 | 26.9 | 18.2 | 38.3 | 17.2 |

**References**

1. Feng X, Park DS, Walker C, Peterson AT, Merow C, Papes M (2019) A checklist for maximizing reproducibility of ecological niche models. Nature Ecology and Evolution 3: 1382–1395.
2. Anciães M, Peterson AT (2009) Ecological niches and their evolution among Neotropical manakins (Aves: Pipridae). Journal of Avian Biology 40: 591–604.
3. Silva SM, Agne CE, Aleixo A, Bonatto AL (2018) Phylogeny and systematics of *Chiroxiphia* and *Antilophia* manakins. Molecular Phylogenetics and Evolution 127: 706–711.
4. Hijmans RJ, Cameron S, Parra J (2005) WorldClim, version 1.3. Retrieved from http://biogeo.berkeley.edu/worldclim/worldclim.htm
5. Hijmans RJ, Guarino L, Bussink C, Mathur P, Cruz M, Barrentes I, et al. (2004) DIVA-GIS. Version 7.1.7. A geographic information system for the analysis of species distribution data. Retrieved from http://www.diva-gis.org/.
6. ESRI (versions 2013, 2014, 2016, 2019) ArcGIS Desktop: Release 10. Redlands, CA: Environmental Systems Research Institute.
7. Phillips S, Anderson R, Schapire R (2006) Maximum entropy modeling of species geographic distributions. Ecological Modelling 190: 231–259.
8. Franklin J (2010) Mapping Species Distributions, Spatial Inference and Prediction. Cambridge University Press. New York, USA.
9. Martin MD, Omland KE (2011) Environmental niche modeling reveals climatic differences among breeding ranges of Orchard Oriole subspecies. The American Midland Naturalist 166: 404–414.
10. Parra JL, Graham CH, Freile JF (2004) Evaluating alternative data sets for ecological niche models of birds in the Andes. Ecography 27: 350–360.
11. Barve N (2008) Tool for Partial-ROC 1.0. Biodiversity Institute, University of Kansas. Lawrence, KS, USA.
12. Peterson AT, Papeş M, Soberón J (2008) Rethinking receiver operating characteristic analysis applications in ecological niche modeling. Ecological Modelling 213: 63–72.
13. R Core Team (2017) R: A Language and Environment for Statistical Computing. R Foundation for Statistical Computing. Vienna, Austria.
14. Barve N, Barve V (2013) ENMGadgets: Tools for Pre and Post Processing in ENM workflow. https://github.com/ vijaybarve/ENMGadgets (accessed December 2017).
15. Merow C, Smith MJ, Silander JA (2013) A practical guide to MaxEnt for modeling species’ distributions: What it does, and why inputs and settings matter. Ecography 36: 1058–1069.
16. U.S. Geological Survey (2016) Digital Elevation Model (DEM) from the Hydrologic Derivatives for Modeling and Applications (HDMA) database - South America. Available at https://www.sciencebase.gov. Accessed January 2016.
